# Characterization of nitrogen compounds in coker gas oil by electrospray ionization Fourier transform ion cyclotron resonance mass spectrometry and Fourier transform infrared spectroscopy

**DOI:** 10.1007/s13203-014-0083-9

**Published:** 2014-09-10

**Authors:** Chen Xiaobo, Liu Yibin, Wang Jin, Shan Honghong, Yang Chaohe, Li Chunyi

**Affiliations:** State Key Laboratory of Heavy Oil Processing, China University of Petroleum, Qingdao, 266580 Shandong People’s Republic of China

**Keywords:** Nitrogen compounds, Coker gas oil, ESI FT-ICR MS, FT-IR, Characterization

## Abstract

In this study, the classes and structures of nitrogen species in coker gas oil (CGO) are characterized by electrospray ionization (ESI) Fourier transform ion cyclotron resonance mass spectrometry (FT-ICR MS) combined with Fourier transform infrared (FT-IR) spectroscopy. The results demonstrate that the *m*/*z* of basic and non-basic nitrogen compounds ranges from 180 to 560 and from 200 to 460, respectively. Six basic nitrogen compounds, N1 (a molecule contains one nitrogen atom, similarly hereinafter), N1O1, N1O1S1, N1O2, N1S1, and N2, are identified by their positive-ion mass spectra, and four non-basic nitrogen compounds, N1, N1O1, N1S1, and N2, are characterized by their negative-ion mass spectra. Among these nitrogen compounds, the N1 class species are the most predominant. Combined with the data of ESI FT-ICR MS and FT-IR, the basic N1 class species are likely pyridines, naphthenic pyridines, quinolines, and benzoquinolines. The most non-basic N1 class species are derivatives of benzocarbazole. The N2 class species are likely amphoteric molecules with pyridine and pyrrole core structures.

## Introduction

Coker gas oil (CGO), a main product derived from the coking process, can be used as the feedstock of fluid catalytic cracking (FCC) unit. However, when the FCC unit is blended with a certain amount (generally less than 20 wt%) of CGO, the activity and selectivity of catalyst decrease, the feedstock conversion drops, recycle oil and slurry oil withdrawal increase, the yield of coke increases, product distribution deteriorates, and CGO bending ratio becomes strictly limited [1–3]. Earlier works [4, 5] on this subject concluded that nitrogen compounds in CGO, especially basic nitrogen compounds, could heavily deactivate FCC catalysts. The poisonous basic nitrogen compounds could reversibly adsorb onto the acid site of catalyst, resulting in a diminution of acid centers, or acted as coke precursors due to their size and aromatic nature [6]. Fu and schaffer [7] and Ho et al. [8] proposed that nitrogen content, proton affinity, and molecular size (or molecular weight) of basic nitrogen compounds were the key factors affecting the performance of FCC. Nevertheless, Li et al. [9, 10] argued that the effect of structure and composition of basic nitrogen compounds was much more obvious than that of nitrogen content. Consequently, the characterizing nitrogen compounds in CGO and investigating the nitrogen compounds how to deactivate FCC catalysts have become research hotspots.

In recent years, electrospray ionization (ESI) coupled with Fourier transform ion cyclotron resonance mass spectrometry (FT-ICR MS) has been widely applied for characterizing nitrogen compounds in crude oil and its distillates [11–15], oil bitumen sands [16], and shale oils [17, 18]. ESI FT-ICR MS can be used to identify the molecular structure of nitrogen compounds with high-molecular-weight and double-bond equivalent (DBE) values. However, amine species could not be distinguished from pyridine or pyrrole derivatives by ESI FT-ICR MS if they have the same DBE values. For instance, ESI FT-ICR MS cannot identify whether nitrogen compounds with a DBE value of 4 are pyridine derivatives or aniline derivatives. Fortunately, Fourier transform infrared (FT-IR) analysis can provide some chemical functional group information to understand the molecular structures of compounds more clearly. In this study, ESI FT-ICR MS combined with FT-IR was used to characterize the nitrogen compounds in CGO, which can give a more accurate characterization of nitrogen compounds in coker gas oil.

## Experimental section

### Feedstock

In this study, CGO containing 0.7 wt% total sulfur and 0.63 wt% total nitrogen was provided by Shengli Petrochemical Refinery, Sinopec Group, China, (Shengli-CGO). Elemental composition was measured using a Vario EL III elemental analyzer (Elementar Co. Ltd., Germany).

### Sample preparation for ESI FT-ICR MS analysis

A total of 10 mg of oil sample was mixed with 10 mL of toluene. A total of 20 mL of the solution mixture was diluted with 1 mL of toluene/methanol (3:17, v/v) solution. Acetic acid was added (5 μL to every 1 mL of sample solution) to ensure efficient ionization for positive ion ESI analysis, and ammonium hydroxide was added (10 μL to every 1 mL of sample solution) for negative ion ESI analysis. The toluene and methanol used were analytical reagent-grade solvents that were distilled twice and kept in glass bottles with ground glass stoppers [14, 15, 17].

### ESI FT-ICR MS analysis

The CGO samples were analyzed by a Bruker apex-ultra FT-ICR MS equipped with a 9.4-T super-conducting magnet at China University of Petroleum (CUP, Beijing, China). The sample solution was infused via an Apollo II electrospray source at a 180 μL/h by a syringe pump. The conditions for positive ion (or negative ion) formation were −4.0 kV (or 3.5 kV) emitter voltage, −4.5 kV (or 4.0 kV) capillary column front end voltage, and 320 V (or 320 V) capillary column end voltage. Ions accumulated for 0.1 s in a hexapole with 2.4 V (or −2.4 V) direct-current voltages and 200 V_p-p_ radio frequency (RF) amplitudes. The optimized mass for quadrupole 1 (Q1) was 200 Da. Hexapoles of the Qh interface were operated at 5 MHz and 200 V_p-p_ RF amplitude, in which ions accumulated for 0.001 s (or 0.01 s). The delay was set to 1.1 ms to transfer the ions to an ICR cell by electrostatic focusing of transfer optics. The ICR was operated at 13 db (or 13.5 db) attenuation, 150 to 800 Da (or 150 to 1,000 Da) mass range, and 4 M acquired data size. The time domain data sets were co-added from 64 data acquisitions [14, 15, 17].

### Fourier transform infrared (FT-IR) analysis

The chemical functional group presented in CGO was analyzed using a Nexus FT-IR spectrometer purchased from Thermol Nicolet Co., Ltd. The film of the sample was created by placing a drop of CGO in KBr plates. The FT-IR spectrum for the sample was obtained at 4 cm^−1^ resolution and collected from 4,000 cm^−1^ to 400 cm^−1^ [17].

## Results and discussion

### ESI FT-ICR MS analysis for nitrogen compounds in Shengli-CGO

**Fig. 1:**
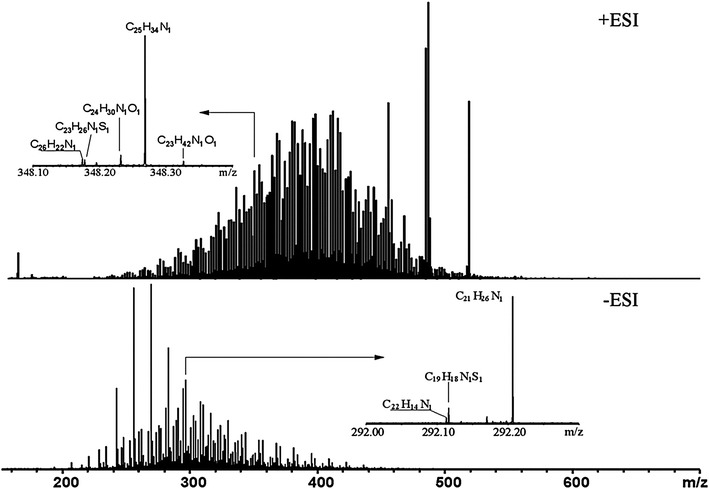
Mass spectra of Shengli-CGO from positive- and negative-ion ESI FT-ICR MS. Insets show the mass scale expanded mass spectra

Figure 1 shows the ESI FT-ICR MS spectra of Shengli-CGO. Positive-ion ESI was used to selectively ionize basic nitrogen compounds (BNC), whereas negative-ion ESI was used for non-basic nitrogen compounds (NBNC). In Fig. 1, the upper part shows the MS spectra of BNC, whereas the lower part shows those of NBNC. The peaks of BNC in Shengli-CGO have a wider mass distribution (180 < *m*/*z* < 560) than NBNC (200 < *m*/*z* < 460). Moreover, BNC has approximately 7,800 peaks in its molecular weight range, whereas NBNC has only 2,100. The distribution peaks of BNC and NBNC are centered approximately at *m*/*z* 390 and 300, respectively. The differences between the positive- and negative-ion ESI FT-ICR mass spectra indicate that BNC in Shengli-CGO has larger average molecular weight, more chemical species, and more complex structures than NBNC.

### Class distribution for nitrogen compounds in Shengli-CGO

**Fig. 2:**
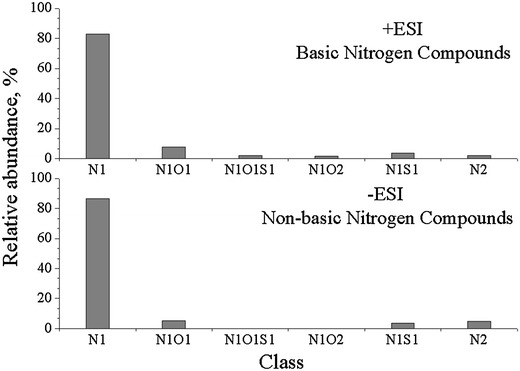
Relative abundance of basic and non-basic nitrogen compounds in Shengli-CGO

The relative abundance of positive- and negative-ion heteroatom class species in Shengli-CGO is shown in Fig. 2. The N1 (a molecule contains one nitrogen atom, similarly hereinafter), N1O1, N1O1S1, N1O2, N1S1, and N2 class species of BNC are identified by positive-ion mass spectra of Shengli-CGO. Among these compounds, the N1 class species are the predominant compounds. The N1, N1O1, N1S1, and N2 class species of NBNC are identified in Shengli-CGO. Similarly, the N1 class species are also the most abundant. Compared with previous research results [14, 15], the class distribution of nitrogen species in Shengli-CGO differ from the CGO samples derived from other Chinese refineries. This finding indicates that heteroatomic compound types are hugely affected by crude oil sources and their storage conditions.

### DBE versus carbon number for nitrogen compounds in Shengli-CGO

**Fig. 3:**
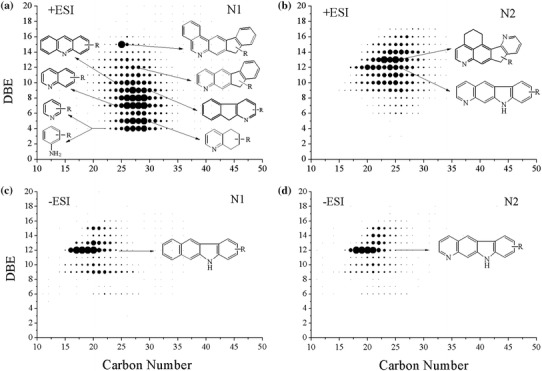
Plots of double-bond equivalents (DBE) versus carbon number for N1 and N2 class species from positive- and negative-ion ESI FT-ICR MS of Shengli-CGO

To identify clearly the molecular structures of nitrogen species in Shengli-CGO, the plots of DBE value versus carbon number for the N1 and N2 class species are presented in Fig. 3. The basic N1 class species distribute a wide range of DBE values (4–20) and carbon numbers (13–45), mainly concentrating at a DBE value range of 4–15 and a carbon number range of 24–31. These N1 class species are most likely derivatives of pyridine, such as pyridines, naphthenic pyridines, quinolines, benzoquinolines, and so on. Their probable structures are shown in Fig. 3a. However, speculating molecular structures according to DBE values is not sufficient. For instance, the core structure of species with a DBE value of 4 could either be pyridines or anilines. Further evidence is needed to confirm this hypothesis. These doubts are further discussed in the subsequent FT-IR analysis section.

The basic N2 class species have DBE values ranging from 6 to 17 and carbon numbers ranging from 17 to 36. The molecular structures of these species are likely amphoteric molecules with a pyridine ring and a pyrrole ring (Fig. 3b). Figure 3c, d shows that the most abundant non-basic N1 and N2 class species have a DBE value of 12. The former species are likely benzocarbazoles and the latter species are similar to the basic N2 class species with pyridine and pyrrole core structures. The DBE values and carbon numbers of BNC are higher than those of NBNC. This finding further proves that BNC has larger average molecular weight, more chemical species, and more complex structures than NBNC.

### FT-IR analysis for Shengli-CGO

**Fig. 4:**
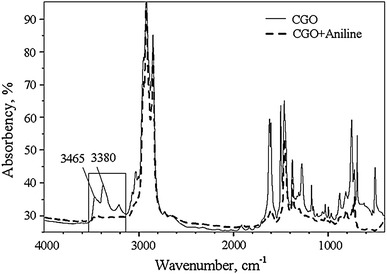
FT-IR spectra of Shengli-CGO and Shengli-CGO + aniline

A Nexus FT-IR spectrometer was used to analyze Shengli-CGO and determine further the molecular structures of nitrogen compounds. The same instrument was also used to measure Shengli-CGO with 1,000 μg g^−1^ of aniline (CGO + aniline) for comparison. The results are shown in Fig. 4. Compared with CGO, the FT-IR spectra of CGO + aniline have obvious absorbance peaks in the range of 3,000 cm^−1^ to 3,500 cm^−1^, which can be attributed to nitrogen–hydrogen (N–H) bonds. Particularly, the absorbance peaks at 3,380 and 3,465 cm^−1^ are assigned to the symmetry stretch mode of the N–H bond in the primary amine, which holds a couple of symmetrical N–H bonds [17]. According to the comparisons, the characteristic absorbance peaks of N–H are not found in CGO. This finding indicates that very few aniline derivatives are found in Shengli-CGO and that the nitrogen atom is almost in the aromatic rings. Therefore, the basic N1 class species with DBE value of 4 in Shengli-CGO are derivatives of pyridine.

## Conclusions

The ESI FT-ICR mass spectra show that the *m*/*z* of BNC and NBNC ranges from 180 to 560 and from 200 to 460, respectively. Six basic nitrogen compounds, N1, N1O1, N1O1S1, N1O2, N1S1, and N2, are identified by positive-ion mass spectra, and four non-basic nitrogen compounds, N1, N1O1, N1S1, and N2, are identified by negative-ion mass spectra. Among these nitrogen compounds in Shengli-CGO, the N1 class species are the most predominant. The FT-IR spectra show that very few aniline derivatives are present in Shengli-CGO and that the nitrogen atom is almost in the aromatic rings. Combined with the data of ESI FT-ICR MS and FT-IR, the chemical structures of the basic N1 class species in Shengli-CGO are likely derivatives of pyridine, such as pyridines, naphthenic pyridines, quinolines, and benzoquinolines. The abundant non-basic N1 class species are derivatives of benzocarbazole. The N2 class species are likely amphoteric molecules with pyridine and pyrrolecore structures.
